# Supplementary material for: Oxytocin and vasotocin receptor variation and the evolution of human prosociality
Source: Compr Psychoneuroendocrinol. 2022 May 5;11:100139. doi: 10.1016/j.cpnec.2022.100139 (PMC9227999; doi:10.1016/j.cpnec.2022.100139)
Supplement: Multimedia component 1 [file mmc1.docx]

**Oxytocin and vasotocin receptor variation and the evolution of human prosociality**

**Supplementary Information**

**Supplementary Note 1: Multialignments**

We list all the multialignments conducted on the OTR, VTR1A and VTR1B sites discussed in the study. Alignments were done using Ortheus^1^, as it is built in the Ensembl^2^ ‘Phylogenetic context’ tool, in order to infer the ancestral alleles of the identified sites. Ortheus is a probabilistic method for the inference of ancestor, whose main contribution is the use of a phylogenetic model incorporating gaps to infer insertion and deletion events. Ancestral sequences are predicted for each node of the phylogenetic tree that relates the sequences. Human (*Homo sapiens*; GRCh38.p13/hg38) sites were searched in Ensembl with their rsIDs, and alignments were made with human as reference. 12 non-human primate species were used to infer the ancestral states: Bonobo (*Pan paniscus*; panpan1.1), Chimpanzee (*Pan troglodytes*; Pan_tro_3.0), Gorilla (*Gorilla gorilla*; gorGor4), Orangutan (*Pongo abelii*; PPYG2), Gibbon (*Nomascus leucogenys*; Nleu_3.0), Vervet (*Chlorocebus sabaeus*; ChlSab1.1), Crab-eating macaque (*Macaca fascicularis*; Macaca_fascicularis_5.0), Macaque (*Macaca mulatta*; Mmul_10), Olive baboon (*Papio Anubis*; Panu_3.0), Gelada (*Theropithecus gelada*; Tgel_1.0), Marmoset (*Callithrix jacchus*; ASM275486v1) and Mouse Lemur (*Microcebus murinus*; Mmur_3.0).

**OTR**

**rs2228485(G/A)**

Human GCACACACGC**G**TTCCCGCTCA

Ancestral sequences 1 GCACACACGC**A**TTCCCGCTCA

Bonobo NNNNNNNNNN**N**NNNNNNNNNN

Ancestral sequences 2 GCACACACGC**A**TTCCCGCTCA

Chimpanzee GCACACACGC**A**TTCCCGCTCA

Ancestral sequences 3 GCACACACGC**A**TTCCCGCTCA

Gorilla GCACACACGC**A**TTCCCGCTCA

Ancestral sequences 4 GCACACACGC**A**TTCCCGCTCA

Orangutan GCACACACGC**A**TTCCCGCTCA

Ancestral sequences 5 GCACACACGC**A**TTCCCGCTCA

Gibbon GCACACACGC**A**TTCCCGCTCA

Ancestral sequences 6 GCACACACGC**A**TTCCCGCTCA

Vervet-AGM GCACACATGC**A**TTCCCGCTCA

Ancestral sequences 7 GCACACACGC**A**TTCCCGCTCA

Crab-eating macaque GCACACACGC**A**TTCCCGCTCA

Ancestral sequences 8 GCACACACGC**A**TTCCCGCTCA

Macaque GCACACACGC**A**TTCCCGCTCA

Ancestral sequences 9 GCACACACGC**A**TTCCCGCTCA

Olive baboon GCACACACGC**A**TTCCCGCTCA

Ancestral sequences 10 GCACACACGC**A**TTCCCGCTCA

Gelada GCACACACGC**A**TTCCCGCTCA

Ancestral sequences 11 GCACACACGC**A**TTCCCGCTCA

Marmoset GCACACACGC**A**TTCCCGCTTA

Ancestral sequences 12 GCACACACGC**A**TTCCCGCTCA

Mouse Lemur GCACGCACGC**G**TTGCCGCCC

**rs237897(A/G)**

Human CCTGCCCACC**A**CTCCTTGCAG

Ancestral sequences 1 CCTGCCCACC**G**CTCCTTGCAG

Bonobo CCTGCCCACC**A**CTCCTTGCAG

Ancestral sequences 2 CCTGCCCACC**G**CTCCTTGCAG

Chimpanzee CCTGCCCACC**G**CTCCTTGCAG

Ancestral sequences 3 CCTGCCCACC**G**CTCCTTGCAG

Gorilla CCTGCCCACC**G**CTCCTTGCAG

Ancestral sequences 4 CCTGCCCACC**G**CTCCTTGCAG

Orangutan CCTGCCCACC**G**CTCCTTGCAG

Ancestral sequences 5 CCTGCCCACC**G**CTCCTTGCAG

Gibbon ACTGCCCACC**G**CTCCTTGCAG

Ancestral sequences 6 CCTGCCCACC**G**CTCCTTGCAG

Vervet-AGM CCTGCCCACC**G**CTTCTTGCAG

Ancestral sequences 7 CCTGCCCACC**G**CTTCTTGCAG

Crab-eating macaque CCTGCCCACC**G**CTTTTTGCAG

Ancestral sequences 8 CCTGCCCACC**G**CTTTTTGCAG

Macaque CCTGCCCACC**G**CTTTTTGCAG

Ancestral sequences 9 CCTGCCCACC**G**CTTCTTGCAG

Olive baboon CCTGCCCACC**A**GTTCTTGCAG

Ancestral sequences 10 CCTGCCCACC**A**CTTCTTGCAG

Gelada CCTGCCCACC**A**CTTCTTGCAG

Ancestral sequences 11 CCTGCCCACC**G**CTCCTTGCAG

Marmoset CCTACCCAGC**G**CTCCCTGCAG

Ancestral sequences 12 CCTGCCCACC**G**CTCCT--CAG

Mouse Lemur CTAGCCCTCA**G**CCCCA--CCG

**rs11131149(A/G)**

Human AAAAAATCGT**G**CTCTAAACCA

Ancestral sequences 1 AAAAAATCGT**G**CTCTAAACCA

Bonobo AAAAAATCGT**G**CTCTAAACCA

Ancestral sequences 2 AAAAAATCGT**G**CTCTAAACCA

Chimpanzee AAAAAATCGT**G**CTCTAAACCA

Ancestral sequences 3 AAAAAATCGT**G**CTCTAAACCA

Gorilla AAAAAATCGT**G**CTGCAAACCA

Ancestral sequences 4 AAA-AATCAT**G**CTCTAAACCA

Orangutan AAA-AATCAT**G**CTCTAAACCA

Ancestral sequences 5 AAA-AATCAT**G**CTCTAAACCA

Gibbon AAA-AATCAT**G**CTCTAAACCA

Ancestral sequences 6 AAA-AATCAT**G**CTCTAAACCA

Vervet-AGM AAA-AATCAT**A**CTCTAAACCA

Ancestral sequences 7 AAA-AATCAT**A**CTCTAAACCA

Crab-eating macaque AAA-AATCAT**A**CTCTAAACCA

Ancestral sequences 8 AAA-AATCAT**A**CTCTAAACCA

[R](https://useast.ensembl.org/Macaca_mulatta/Variation/Compara_Alignments?db=core;vdb=variation;vf=10812112)

Macaque AAA-AATCAT**A**CTCTAAACCA

Ancestral sequences 9 AAA-AATCAT**A**CTCTAAACCA

Olive baboon AAA-AATCAT**A**CTCTAAACCA

Ancestral sequences 10 AAA-AATCAT**A**CTCTAAACCA

Gelada AAA-AATCAT**A**CTCTAAACCA

**rs59190448(G/A)**

Human TGCCATCAGC**G**GATGTGTACC

Ancestral sequences 1 TGCCATCAGC**A**GATGTGTACC

Bonobo TGCCATCAGC**A**GATGTGTACC

Ancestral sequences 2 TGCCATCAGC**A**GATGTGTACC

Chimpanzee TGCCATCAGC**A**GATGTGTACC

Ancestral sequences 3 TGCCATCAGC**A**GATGTGTACC

Gorilla TGCCATCAGC**A**GATGTGTACC

Ancestral sequences 4 TGCCATCAGC**A**GATGTGTACC

Orangutan TGCCATCAGC**A**GATGTGTACC

Ancestral sequences 5 TGCCATCAGC**A**GATGTGTACC

Gibbon TGCCATCAGC**A**GATGTGTACC

Ancestral sequences 6 TGCCATCAGC**A**GATGTGTACC

Vervet-AGM TGCCATCAGC**A**GATGTGTACC

Ancestral sequences 7 TGCCATCAGC**A**GATGTGTACC

Crab-eating macaque TGCCATCAGC**A**GATGTGTACC

Ancestral sequences 8 TGCCATCAGC**A**GATGTGTACC

Macaque TGCCATCAGC**A**GATGTGTACC

Ancestral sequences 9 TGCCATCAGC**A**GATGTGTACC

Olive baboon TGCCATCAGC**A**GATGTGTACC

Ancestral sequences 10 TGCCATCAGC**A**GATGTGTACC

Gelada TGCCATCAGC**A**GATGTGTACC

**rs13316193(C/T)**

Human ACGGGAATGC**T**GTTAAATATC

Ancestral sequences 1 ACGGGAATGC**T**GCTAAATATC

Bonobo ACGGGAATGC**T**GCTAAATATC

Ancestral sequences 2 ACGGGAATGC**T**GCTAAATATC

Chimpanzee AGGGGAATGC**T**GCTAAATATC

Ancestral sequences 3 ACGGGAATGC**T**GCTAAATATC

Gorilla ATGGGAATGC**T**GCTAAATATC

Ancestral sequences 4 ACAGGAATGC**T**GCTAAATATC

Orangutan ACAGGAATGC**T**GCTAAATATC

Ancestral sequences 5 ACAGGAATGC**T**GCTAAATATC

Gibbon ACAGGAATGC**T**GCTGAATATC

Ancestral sequences 6 ACAGGAATGC**T**GCTAAATATC

Vervet-AGM ACAGGAATGC**T**GCTAAGTATC

Ancestral sequences 7 ACAGGAATGC**T**GCTAAATATC

Crab-eating macaque ACAGGAATGC**T**GCTAAATATC

Ancestral sequences 8 ACAGGAATGC**T**GCTAAATATC

Macaque ACAGG[A](https://useast.ensembl.org/Macaca_mulatta/ZMenu/TextSequence?db=core;factorytype=Location;r=3:8760557-8761557;vdb=variation;vf=10812111)ATGC**T**GCTAAATATC

Ancestral sequences 9 ACAGGAATGC**T**GCTAAATATC

Olive baboon ACAGGAATGC**T**GCTAAATATC

Ancestral sequences 10 ACAGGAATGC**T**GCTAAATATC

Gelada ACAGGAATGC**T**GCTAAATATC

Ancestral sequences 11 ACAGGAATGC**T**GCTAAATATC

Marmoset ACAGGAATGC**T**GCTAAATATC

**rs9872310(G/A)**

Human CCGTAAGTAT**A**AGTGTTCATA

Ancestral sequences 1 CCGTAAGTAT**A**AGTGTTCATA

Bonobo CCGTAAGTAT**A**AGTGTTCACA

Ancestral sequences 2 CCGTAAGTAT**A**AGTGTTCATA

Chimpanzee CCGTAAGTAT**A**AGTGTTCATA

Ancestral sequences 3 CCGTAAGTAT**A**AGTGTTCATA

Gorilla CCGTAAGTAT**A**AGTGTTCATA

Ancestral sequences 4 CCATAAGTAT**A**AGTGTTCATA

Orangutan CCATAAGTAT**A**AGTGTTCATA

Ancestral sequences 5 CCATAAGTAT**A**AGTGTTCATA

Gibbon CCATAAGTAT**A**AGTGTTCATG

Ancestral sequences 6 CCATAAGTAT**A**AGTGTTCATA

Vervet-AGM CCATAAGTAT**A**AGTGTTCATA

Ancestral sequences 7 CCATAAGTAT**A**AGTGTTCATA

Crab-eating macaque CCATAAGTAT**A**AGTGTTCATA

Ancestral sequences 8 CCATAAGTAT**A**AGTGTTCATA

Macaque CCATAAGTAT**A**AGTGTTCATA

Ancestral sequences 9 CCATAAGTAT**A**AGTGTTCATA

Olive baboon CCATAAGTAT**A**AGTGTTCATA

Ancestral sequences 10 CCATAAGTAT**A**AGTGTTCATA

Gelada CCATAAGTAT**A**AGTGTTCATA

Ancestral sequences 11 CCATAAGTAT**A**AGTGTTCATA

Marmoset CCATAAGTAC**A**AGTGTTCATA

Ancestral sequences 12 CCATAAGTAT**A**AGTGTTCATA

Mouse Lemur CCATAAATGT**A**GGTGTTCGTA

**rs4686302(T/C)**

Human CCGTAGCAGG**C**AGCGAGCACG

Ancestral sequences 1 CCGTAGCAGG**C**AGCGAGCACG

Bonobo CCGTAGCAGG**C**AGCGAGCACG

Ancestral sequences 2 CCGTAGCAGG**C**AGCGAGCACG

Chimpanzee CCGTAGCAGG**C**AGCGAGCACG

Ancestral sequences 3 CCGTAGCAGG**C**AGCGAGCACG

Gorilla CCGTAGCAGG**C**AGCGAGCACG

Ancestral sequences 4 CCGTAGCAGG**C**AGCGAGCACG

Orangutan CCGTAGCAGG**C**AGCGAGCACG

Ancestral sequences 5 CCGTAGCAGG**C**AGCGAGCACG

Gibbon CCGTAGCAGG**C**AGCGAGCACG

Ancestral sequences 6 CCGTAGCAGG**C**AGCGAGCACG

Vervet-AGM CCATAGCAGG**C**AGCGAGTACG

Ancestral sequences 7 CCATAGCAGG**C**AGCGAGCACG

Crab-eating macaque CCATAGCAGG**C**AGCGAGCACG

Ancestral sequences 8 CCATAGCAGG**C**AGCGAGCACG

Macaque CCATAGCAGG**C**AGCGAGCACG

Ancestral sequences 9 CCATAGCAGG**C**AGCGAGCACG

Olive baboon CCATAGCAGG**C**AGCGAGCACG

Ancestral sequences 10 CCATAGCAGG**C**AGCGAGCACG

Gelada CCATAGCAGG**C**AGCGAGCACG

Ancestral sequences 11 CCGTAGCAGG**C**GGCTAGCACG

Marmoset CCGTAGCAGG**C**GGCTAGCATG

Ancestral sequences 12 CCGTAGCAGG**C**GGCCAGCACG

Mouse Lemur CCGTAGCAGG**C**GGCCAGCACG

**rs237888(T/C)**

Human CCTAGTTGGA**T**ACAGTTATTT

Ancestral sequences 1 CCTAGTTGGA**C**ACAGTTATTT

Bonobo CCTAGTTGGA**C**ACAGTTATTT

Ancestral sequences 2 CCTAGTTGGA**C**ACAGTTATTT

Chimpanzee CCTAGTTGGA**C**ACAGTTATTT

Ancestral sequences 3 CCTAGTTG--**-**----------

Gorilla CCTAGTTG--**-**----------

Ancestral sequences 4 CCTAGTTG--**-**----------

Orangutan CCTAGTTG--**-**----------

Ancestral sequences 5 CCTAGTTG--**-**----------

Gibbon CCTAGTTG--**-**----------

Ancestral sequences 6 CCTAGTTG--**-**----------

Vervet-AGM CCTAGTTG--**-**----------

Ancestral sequences 7 CCTAGTTG--**-**----------

Crab-eating macaque CCTAGTTG--**-**----------

Ancestral sequences 8 CCTAGTTG--**-**----------

Macaque CCTAGTTG--**-**----------

Ancestral sequences 9 CCTAGTTG--**-**----------

Olive baboon CCTAGTTG--**-**----------

Ancestral sequences 10 CCTAGTTG--**-**----------

Gelada CCTAGTTG--**-**----------

**rs60902022(C/T)**

Human CTTCAAAGGA**T**GGGAATCCGT

Ancestral sequences 1 CTTCAAAGGA**T**GGGAATCCGT

Bonobo CTTCAAAGGA**T**GGGAATCCGT

Ancestral sequences 2 CTTCAAAGGA**T**GGGAATCCGT

Chimpanzee CTTCAAAGGA**T**GGGAATCCGT

Ancestral sequences 3 CTTCAAAGGA**T**GGGAATCCGT

Gorilla CTTCAAAGGA**T**GGGAATCCGT

Ancestral sequences 4 CTTCAAAGGA**T**GGGAATCCAT

Orangutan CTTCAAAGGA**T**GGGAATCCAT

Ancestral sequences 5 CTTCAAAGGA**T**GGGAATCCAT

Gibbon CTTCAAAGGA**T**GGGAATCCAT

Ancestral sequences 6 CTTCAAAGGA**T**GGGAATCCAT

Vervet-AGM CTTCAAAGGA**T**GGGAATCCAT

Ancestral sequences 7 CTTCAAAGGA**T**GGGAATCCAT

Crab-eating macaque CTTCAAAGGA**T**GGGAATCCAT

Ancestral sequences 8 CTTCAAAGGA**T**GGGAATCCAT

Macaque CTTCAAAGGA**T**GGGAATCCAT

Ancestral sequences 9 CTTCAAAGGA**T**GGGAATCCAT

Olive baboon CTTCAAAGGA**T**GGGAATCCAC

Ancestral sequences 10 CTTCAAAGGA**T**GGGAATCCAT

Gelada CTTCAAAGGA**T**GGGAATCCAT

Ancestral sequences 11 CTTCAAAGGA**T**GGGAATCCAT

Marmoset CTTCAAGGGA**T**GGGAATCCAT

**rs6770632(C/A)**

Human AATTTCTTTC**C**AATTTT-------------GTAG

Ancestral sequences 1 AATTTCTTTC**A**AATTTT-------------GTAG

Bonobo AATTTCTTTC**A**AATTTT-------------GTAG

Ancestral sequences 2 AATTTCTTTC**A**AATTTT-------------GTAG

Chimpanzee AATTTCTTTC**A**AATTTT-------------GTAG

Ancestral sequences 3 AATTTCTTTC**A**AATTTT-------------GTAG

Gorilla AATTTCTTTC**A**AATTTT-------------GTAG

Ancestral sequences 4 AATTTCTTTC**A**AATTTT-------------GTAG

Orangutan AATTTCTTTC**A**AATTTT-------------GTAG

Ancestral sequences 5 AATTTCTT--**-**-------------------GTAG

Gibbon AATTTCTT--**-**-------------------GTAG

Ancestral sequences 6 AATTTCTT--**-**------CCAAA----TTTTGTAG

Vervet-AGM AATTTCTT--**-**------CCAAA----TTTTGTAG

Ancestral sequences 7 AATTTCTT--**-**------CCAAA----TTTTGTAG

Crab-eating macaque AATTTCTT--**-**------CCAAA----TTTTGTAG

Ancestral sequences 8 AATTTCTT--**-**------CCAAA----TTTTGTAG

Macaque AATTTCTT--**-**------CCAAA----TTTTGTAG

Ancestral sequences 9 AATTTCTT--**-**------CCAAA----TTTTGTAG

Olive baboon AATTTCTT--**-**------CCAAA----TTTTGTAG

Ancestral sequences 10 AATTTCTT--**-**------CCAAA----TTTTGTAG

Gelada AATTTCTT--**-**------CCAAA----TTTTGTAG

Ancestral sequences 11 AATTTCTT--**-**------TCAAA----TTTTGTAG

Marmoset AATTTCTT--**-**------TCAAA----TTTTGTAG

Ancestral sequences 12 AATTTCTT--**-**------TCAAA----TTTTGTAG

Mouse Lemur AATTTCTT--**-**------TCAATAGTAGGTTGTAG

**rs237885(T/G)**

Human CATCTTGTGG**T**TTAGGTAGGC

Ancestral sequences 1 CATCTTGTGG**G**TTAGGTAGGC

Bonobo CATCTTGTGG**G**TTAGGTAGGC

Ancestral sequences 2 CATCTTGTGG**G**TTAGGTAGGC

Chimpanzee CATCTTGTGG**G**TTAGGTAGGC

Ancestral sequences 3 CATCTTGTGG**G**TTAGGTAGGC

Gorilla CATCTTGTGG**G**TTAGGTAGGC

Ancestral sequences 4 CATCTTGTGG**G**TTAGGTAGGC

Orangutan CATCTTA[C](https://useast.ensembl.org/Pongo_abelii/ZMenu/TextSequence?db=core;factorytype=Location;r=3:8753357-8754357;vdb=variation;vf=7291215)GG**G**TTAGGTAGGC

Ancestral sequences 5 CATCTTGTGG**G**TTAGGTAGGC

Gibbon CATCTTGTGG**G**TTAGGTAGGC

Ancestral sequences 6 CATCTTGTGG**G**TTAGGTAGGC

Vervet-AGM CATCTTGTGA**G**TTACGTAGGC

Ancestral sequences 7 CATCTTGTGA**G**TTAGGTAGGC

Crab-eating macaque CATCTTGTGG**G**TTAGGTAGGC

Ancestral sequences 8 CATCTTGTGA**G**TTAGGTAGGC

Macaque CATCTTGTG[A](https://useast.ensembl.org/Macaca_mulatta/ZMenu/TextSequence?db=core;factorytype=Location;r=3:8753357-8754357;vdb=variation;vf=10811958)**G**TTAGGTAGGC

Ancestral sequences 9 CATCTTGTGA**G**TTAGGTAGGC

Olive baboon CATCTTGTGA**G**TTAGGTAGTC

Ancestral sequences 10 CATCTTGTGA**G**TTAGGTAGTC

Gelada CATCTTGTGA**G**TTAGGTAGTC

Ancestral sequences 11 CATCTTGTGG**G**TTAGGTAGGC

Marmoset CATCTTGTGG**G**TTAGGTAGGC

Ancestral sequences 12 ---------G**G**TTAGGTAGGC

Mouse Lemur ---------G**G**CTGGGCGGGG

**rs1042778(G/T)**

Human CCCCAAGGAG**G**GGAGGGATAC

Ancestral sequences 1 CCCCAAGGAG**T**GGAGGGATAC

Bonobo CCCCAAGGAG**T**GGAGGGATAC

Ancestral sequences 2 CCCCAAGGAG**T**GGAGGGATAC

Chimpanzee CCCCAAGGAG**T**GGAGGGATAC

Ancestral sequences 3 CCCCAAGGAG**T**GGAGGGATAC

Gorilla CCCCAAGTTG**T**GGAGGGATAT

Ancestral sequences 4 CCCCAAGGAG**T**GGAGGGATAC

Orangutan CCCCAAGGAG**T**GGAGGGATAC

Ancestral sequences 5 CCCCAAGGAG**T**GGAGGGATAC

Gibbon CCCCAAGGAG**T**GGAGGGATAC

Ancestral sequences 6 CCCCAAGGAG**T**GGAGGGATAC

Vervet-AGM CCCCAAGGAG**T**GGAGGGAAAC

Ancestral sequences 7 CCCCAAGGAG**T**GGAGGGATAC

Crab-eating macaque CCCCAAGGAG**T**GGAGGGATAC

Ancestral sequences 8 CCCCAAGGAG**T**GGAGGGATAC

Macaque CCCCAAGGAG**T**[A](https://useast.ensembl.org/Macaca_mulatta/ZMenu/TextSequence?db=core;factorytype=Location;r=3:8752359-8753359;vdb=variation;vf=10811940)GAGGGATAC

Ancestral sequences 9 CCCCAAGGAG**T**GGAGGGATAC

Olive baboon CCCCAAGGAG**T**GGAGGGATAC

Ancestral sequences 10 CCCCAAGGAG**T**GGAGGGATAC

Gelada CCCCAAGGAG**T**GGAGGGATAC

Ancestral sequences 11 CCCCAAGGAG**T**GGAGGGATAC

Marmoset CCCCAAGGAG**T**GGAGCGATAC

Ancestral sequences 12 CCCCAAGGAG**T**GGAGGGATAC

Mouse Lemur CCCCCAGGAG**C**GGAGGCATTC

**rs237911(G/A)**

Human GGATCTGCTG**G**GTCC-ACCCTG

Ancestral sequences 1 GGATCTGCTG**A**GTCC-ACCCTG

Bonobo GGATCTGCTG**A**GTCC-ACCCTG

Ancestral sequences 2 GGATCTGCTG**A**GTCC-ACCCTG

Chimpanzee GGATCTGCTG**A**GTCC-ACCCTG

Ancestral sequences 3 GGATCTGCTG**A**GTCC-ACCCTG

Gorilla GGATCTGCTG**A**GTCC-ACCCTG

Ancestral sequences 4 GGATCTGCTG**G**GTCC-ACCCTG

Orangutan GGATCTGCTG**G**GTCC-ACCCTG

Ancestral sequences 5 GGATCTGCTG**G**GTCC-ACCCTG

Gibbon GGATCTGCTG**G**GTCC-ACCCTG

Ancestral sequences 6 GGATCTGCTG**G**GTCC-ACCCTG

Vervet-AGM GGATCTGCTG**G**GTCC-ACCCTG

Ancestral sequences 7 GGATCTGCTG**G**GTCC-ACCCTG

Crab-eating macaque GGATCTGCTG**G**GTCC-ACCCTG

Ancestral sequences 8 GGATCTGCTG**G**GTCC-ACCCTG

Macaque GGATCTGCTGGGTCC-ACCCTG

Ancestral sequences 9 GGATCTGCTG**G**GTCC-ACCCTG

Olive baboon GGATCTGCTG**G**GTCC-ACCCTG

Ancestral sequences 10 GGATCTGCTG**G**GTCC-ACCCTG

Gelada GGATCTGCTG**G**GTCC-ACCCTG

Ancestral sequences 11 GGATCTGCTG**G**GTCC-ACCCTG

Marmoset AGATCTGCTG**G**GTCT-ACCCTG

Ancestral sequences 12 GGATCTGCTG**G**GTCC-ACCCTG

Mouse Lemur GGCTCTGCCG**G**GTCCCAGCCTG

**rs2254298(A/G)**

Human CCG-CAAACTG**G**GAAAACAG--GG

Ancestral sequences 1 CCG-CAAACTG**G**GAAAACAG--GG

Bonobo CCG-CAAACTG**G**GAAAACAG--GG

Ancestral sequences 2 CCG-CAAACTG**G**GAAAACAG--GG

Chimpanzee CCG-CAAACTG**G**GAAAACAG--GG

Ancestral sequences 3 CCG-CAAACTG**G**GAAAACAG--GG

Gorilla CCA-CAAACTG**G**GAAAACAG--GG

Ancestral sequences 4 CCG-CAAACTG**G**GAAAACAG--GG

Orangutan CCG-CAAACTG**G**GAAAACAG--GG

Ancestral sequences 5 CCG-CAAACTG**G**GAAAACAG--GG

Gibbon CCG-CAAACTG**G**GAAAACAG--GG

Ancestral sequences 6 CCTCCAAACTG**G**GAAAACAG----

Vervet-AGM CCTCCAAAGTG**G**GAAAACAG----

Ancestral sequences 7 CCTCCAAAGTG**G**GAAAACAG----

Crab-eating macaque CCTCCAAAGTG**G**GAAAACAGGG--

Ancestral sequences 8 CCTCCAAAGTG**G**GAAAACAGGG--

[R](https://useast.ensembl.org/Macaca_mulatta/Variation/Compara_Alignments?db=core;vdb=variation;vf=10812097)

Macaque CCTCCAA[A](https://useast.ensembl.org/Macaca_mulatta/ZMenu/TextSequence?db=core;factorytype=Location;r=3:8760042-8761042;vdb=variation;vf=10812097)GTG**G**GAAAACAGGG--

Ancestral sequences 9 CCTCCAAAGTG**G**GAAAACAGGG--

Olive baboon CCTCCAAAGTG**G**GAAAACAGGG--

Ancestral sequences 10 CCTCCAAAGTG**G**GAAAACAGGG--

Gelada CCTCCAAAGTG**G**GAAAACAGGG--

Ancestral sequences 11 CCTCCAAACTG**G**GAAAACAG----

Marmoset CCCCTAAACTG**G**AAAATCAG----

**rs53576(A/G)**

Human ATGCCCGAGG**A**TCCTCAGTCC

Ancestral sequences 1 ATGCCCGAGG**G**TCCTCAGTCC

Bonobo ATGCCCGAGG**G**TCCTCAGTCC

Ancestral sequences 2 ATGCCCGAGG**G**TCCTCAGTCC

Chimpanzee ATGCCCGAGG**G**TCCTCAGTCC

Ancestral sequences 3 ATGCCCGAGG**G**TCCTCAGTCC

Gorilla ATGCCCGAGG**G**TCCTCAGTCC

Ancestral sequences 4 ATGCCCGAGG**G**TCCTCAGTCC

Orangutan ATGCCCGAGG**G**TCCTCAGTCC

Ancestral sequences 5 ATGCCAGAGG**G**TCCTCAGTCC

Gibbon ATGCCAGAGG**G**TCCTCAGTCC

Ancestral sequences 6 ATGCCAGAGG**G**TCCTCAGTCC

Vervet-AGM ATGCCAGATG**G**TCCTCAGTCC

Ancestral sequences 7 ATGCCAGATG**G**TCCTCAGTCC

Crab-eating macaque ATGCCAGATG**G**TCCTCAGTCC

Ancestral sequences 8 ATGCCAGATG**G**TCCTCAGTCC

Macaque ATGCCAGATG**G**TCCTCAGTCC

Ancestral sequences 9 ATGCCAGATG**G**TCCTCAGTCC

Olive baboon ATGCCAGATG**G**TCCTCAGTCC

Ancestral sequences 10 ATGCCAGATG**G**TCCTCAGTCC

Gelada ATGCCAGATG**G**TCCTCAGTCC

Ancestral sequences 11 ATGCCAGAGG**G**TCCTCAGTCC

Marmoset GTGCCAGAGG**A**TCCTCAGTCC

**rs2268490(T/C)**

Human CACTG--TTTTG**C**CTAGTTGGAT

Ancestral sequences 1 CACTG--TTTTG**C**CTAGTTGGAC

Bonobo CACTG--TTTTG**C**CTAGTTGGAC

Ancestral sequences 2 CACTG--TTTTG**C**CTAGTTGGAC

Chimpanzee CACTG--TTTTG**C**CTAGTTGGAC

Ancestral sequences 3 CACTGTGTTTTG**C**CTAGTTG---

Gorilla CACTGTGTTTTG**C**CTAGTTG---

Ancestral sequences 4 CACTGTGTTTTG**C**CTAGTTG---

Orangutan CACTGTGTTTTG**C**CTAGTTG---

Ancestral sequences 5 CACTGTGTTTTG**C**CTAGTTG---

Gibbon CACTGAGTTTTG**C**CTAGTTG---

Ancestral sequences 6 CACTGTGTTTTG**C**CTAGTTG---

Vervet-AGM CACCGTGTTTTG**C**CTAGTTG---

Ancestral sequences 7 CACCGTGTTTTG**C**CTAGTTG---

Crab-eating macaque CACCGTGTTTTG**C**CTAGTTG---

Ancestral sequences 8 CACCGTGTTTTG**C**CTAGTTG---

Macaque CACCGTGTTTTG**C**CTAGTTG---

Ancestral sequences 9 CACCGTGTTTTG**C**CTAGTTG---

Olive baboon CACCGTGTTTTG**C**CTAGTTG---

Ancestral sequences 10 CACCGTGTTTTG**C**CTAGTTG---

Gelada CACCGTGTTTTG**C**CTAGTTG---

**rs2268493(C/T)**

Human AAGAAATGAA**T**AAAGTAACTG

Ancestral sequences 1 AAGAAATGAA**T**AAAGTAACTG

Bonobo AAGAAATGAA**T**AATGTCACTG

Ancestral sequences 2 AAGAAATGAA**T**AATGTCACTG

Chimpanzee AAGAAATGAA**T**AATGTCACTG

Ancestral sequences 3 AAGAAATGAA**T**AAAGTAACTG

Gorilla AAGAAATGAA**T**AAAGTAACTG

Ancestral sequences 4 AAGAAATGAA**T**AAAGTAACTG

Orangutan AAGAAATGAA**T**AAAGTAACTG

Ancestral sequences 5 AAGAAATGAA**T**AAAGTAACTG

Gibbon AAGAAATGAA**T**GAAGTAACTG

Ancestral sequences 6 AAGAAATGAA**T**AAAGTAACTG

Vervet-AGM AAGAAATGAA**T**AAAGTAACCG

Ancestral sequences 7 AAGAAATGAA**T**AAAGTAACCG

Crab-eating macaque AAGAAATGAA**T**AAAGTAACCA

Ancestral sequences 8 AAGAAATGAA**T**AAAGTAACCG

Macaque AAGAAATGAA**T**AAAGTAACCG

Ancestral sequences 9 AAGAAATGAA**T**AAAGTAACCG

Olive baboon AAGAAATGAA**T**AAAGTAACCA

Ancestral sequences 10 AAGAAATGAA**T**AAAGTAACCG

Gelada AAGAAATGAA**T**AAAGTAACCG

Ancestral sequences 11 AAGAAATGAA**T**AAAGTAACTG

Marmoset AAGAAATGAA**T**AAAGTAACTG

**rs237917(T/C)**

Human CTAACTTAGT**T**TTAATCTAAA

Ancestral sequences 1 CTAATTTAGT**C**TTAATCTAAA

Bonobo CTAATTTAGT**C**TTAATCTAAA

Ancestral sequences 2 CTAATTTAGT**C**TTAATCTAAA

Chimpanzee CTAATTTAGT**C**TTAATCTAAA

Ancestral sequences 3 CTAATTTAGT**C**TTAATCTAAA

Gorilla CTAATTTAGT**C**TTAATCTAAA

Ancestral sequences 4 CTAATTTAGT**C**TTAATCTAAA

Orangutan CTAATTTAGT**C**TTAATCTAAA

Ancestral sequences 5 CTAATTTAGT**C**TTAATCTAAA

Gibbon CTAATTTAGT**C**TTAATCTAAA

Ancestral sequences 6 CTAATTTAGT**C**TTAATCTAAA

Vervet-AGM CTAATTTAGT**C**TTAATCTAAA

Ancestral sequences 7 CTAATTTAGT**C**TTAATCTAAA

Crab-eating macaque CTAATTTAGT**C**TTAATCTAAA

Ancestral sequences 8 CTAATTTAGT**C**TTAATCTAAA

Macaque CTAATTTAGT**C**TTAATCTAAA

Ancestral sequences 9 CTAATTTAGT**C**TTAATCTAAA

Olive baboon CTAATTTAGT**C**TTAATCTAAA

Ancestral sequences 10 CTAATTTAGT**C**TTAATCTAAA

Gelada CTAATTTAGT**C**TTAATCTAAA

Ancestral sequences 11 CTAATTTAGC**C**TTAATCTTAA

Marmoset CTAATTCAGC**C**TTGATCTTAA

Ancestral sequences 12 CTAATTTAGC**C**TTAATCTTAA

Mouse Lemur CTAATTTGGC**C**TTCCTCTTAA

**rs237889(T/C)**

Human AGCAAGGCCA**T**AGGAACTTGT

Ancestral sequences 1 AGCAAGGCCA**C**AGGAACTTGT

Bonobo AGCAAGGCCA**C**AGGAACTTGT

Ancestral sequences 2 AGCAAGGCCA**C**AGGAACTTGT

Chimpanzee AGCAAGGCCA**C**AGGAACTTGT

Ancestral sequences 3 AGCAAGGCCA**C**AGGAACTTGT

Gorilla AGCAAGGCCA**C**AGGAACTTGT

Ancestral sequences 4 AGCAAGGCCA**C**AGGAACTTGT

Orangutan AGCAAGGCCA**C**AGGAACTTGT

Ancestral sequences 5 AGCAAGGCCA**C**AGGAACTTGT

Gibbon GGCAAGGCCA**C**AGGAACTTGT

Ancestral sequences 6 AGCAAGGCCA**C**AGGAACTTGT

Vervet-AGM AGCAAGGCCA**C**AGGAACTTGT

Ancestral sequences 7 AGCAAGGCCA**C**AGGAACTTGT

Crab-eating macaque AGCAAGGCCA**C**AGGAACTTGT

Ancestral sequences 8 AGCAAGGCCA**C**AGGAACTTGT

Macaque AGCAAGGCCA**C**AGGAACTTGT

Ancestral sequences 9 AGCAAGGCCA**C**AGGAACTTGT

Olive baboon AGCAAGGCCA**C**AGGAACTTGT

Ancestral sequences 10 AGCAAGGCCA**C**AGGAACTTGT

Gelada AGCAAGGCCA**C**AGGAACTTGT

Ancestral sequences 11 AGCAAGGCCA**C**AGGAACTTGT

Marmoset AGCAAAGCCA**C**AGGAGCTGGT

**VTR1A**

**rs1042615(A/G)**

Human AGGCCGACGC**A**-AACATGCCGA

Ancestral sequences 1 AGGCCGACGC**G**-AACATGCCGA

Bonobo AGGCCGACGC**G**-AACATGCCGA

Ancestral sequences 2 AGGCCGACGC**G**-AACATGCCGA

Chimpanzee AGGCCGACGC**G**-AACATGCCGA

Ancestral sequences 3 AGGCCGACGC**G**-AACATGCCGA

Gorilla AGGCCGACGC**G**-AACATGCCGA

Ancestral sequences 4 AGGCCGACGC**G**-AACATGCCGA

Orangutan AGGCCGACGC**G**-AACATGCCGA

Ancestral sequences 5 AGGCCGACGC**G**-AACATGCCGA

Gibbon AGGCCGACGC**G**AAACATGCCGA

Ancestral sequences 6 AGGCCGACGC**G**-AACATGCCGA

Vervet-AGM AGGCCGACGC**G**-AACATGCCGA

Ancestral sequences 7 AGGCCGACGC**G**-AACATGCCGA

Crab-eating macaque AGGCCGACGC**G**-AACATGCCGA

Ancestral sequences 8 AGGCCGACGC**G**-AACATGCCGA

Macaque AGGCCGACGC**G**-AACATGCCGA

Ancestral sequences 9 AGGCCGACGC**G**-AACATGCCGA

Olive baboon AGGCCGACGC**G**-AACATGCCGA

Ancestral sequences 10 AGGCCGACGC**G**-AACATGCCGA

Gelada AGGCCGACGC**G**-AACATGCCGA

Ancestral sequences 11 AGGCCGACGC**G**-AACATGCCGA

Marmoset AGGCCGATGC**G**-AACATGCCAA

Ancestral sequences 12 AGGCCGACGC**G**-AACATGCCGA

Mouse Lemur AGGCCGACGC**G**-AACATGCCGA

**rs3803107(A/G)**

Human GAAAATAAAA**G**-----AAACTAACAA

Ancestral sequences 1 GAAAATAAAA**G**-----AAACCAACAA

Bonobo GAAAATAAAA**G**-----AAACCAACAA

Ancestral sequences 2 GAAAATAAAA**G**-----AAACCAACAA

Chimpanzee GAAAATAAAA**G**-----AAACCAACAA

Ancestral sequences 3 GAAAATAAAA**G**-----AAACCAACAA

Gorilla GAAAATAAAA**G**-----AAACCAACAA

Ancestral sequences 4 GAAAATAAAA**G**-----AAACCAACAA

Orangutan GAAAATAAAA**G**-----AAACCAACAA

Ancestral sequences 5 GAAAATAAAA**G**-----AAACCAACAA

Gibbon GAAAATAAAA**G**-----AAACCAACAA

Ancestral sequences 6 GAAAATAAAA**G**-----AAACCAACAA

Vervet-AGM GAAAATAAAA**G**-----AAACCAACAA

Ancestral sequences 7 GAAAATAAAA**G**-----AAACCAACAA

Crab-eating macaque GAAAAT----**-**-----AAACCAACAA

Ancestral sequences 8 GAAAAT----**-**-----AAACCAACAA

Macaque GAAAAT----**-**-----AAACCAACAA

Ancestral sequences 9 GAAAAT----**-**-----AAACCAACAA

Olive baboon GAAAAT----**-**AAAAGAAACCAACAA

Ancestral sequences 10 GAAAAT----**-**AAAAGAAACCAACAA

Gelada GAAAAT----**-**AAAAGAAACCAACAA

Ancestral sequences 11 GAAAATAAAA**G**-----AAACCAACAA

Marmoset GAAAATAAAA**G**-----AAACCAATAA

Ancestral sequences 12 GAAAATAAAA**G**-----AAACCAACAA

Mouse Lemur AAAATTAAAA**G**-----AAATCAACAA

**rs10784339(C/G)**

Human A-AATACAACT**G**GGTAGGGTGA

Ancestral sequences 1 A-AATACAACT**G**GGTAGGGTGA

Bonobo A-AATACGACT**G**GGTAGGGTGA

Ancestral sequences 2 A-AATACAACT**G**GGTAGGGTGA

Chimpanzee A-AATACAACT**G**GGTAGGGTGA

Ancestral sequences 3 A-AATACAACT**G**GGTAGGGTGA

Gorilla A-AATACAACT**G**GGTAGGGTGA

Ancestral sequences 4 A-AATACAACT**G**GGTAGGGTGA

Orangutan A-AATACAACT**G**GGTAGAGTGA

Ancestral sequences 5 A-AATACAACT**G**GGTAGGGTGA

Gibbon A-AATACAGCA**G**GGTAGGGTGA

Ancestral sequences 6 A-AATACAACT**G**GGTAGGGTGA

Vervet-AGM A-AAAACAACT**G**GGTAGGGTGA

Ancestral sequences 7 A-AAAACAACT**G**GGTAGGGTGA

Crab-eating macaque A-AAAACAACT**G**GGTAGGGTGA

Ancestral sequences 8 A-AAAACAACT**G**GGTAGGGTGA

Macaque AAAAAACAACT**G**GGTAGGGTGA

Ancestral sequences 9 A-AAAACAACT**G**GGTAGGGTGA

Olive baboon A-AAAACAACT**G**GGTAGGGTGA

Ancestral sequences 10 A-AAAACAACT**G**GGTAGGGTGA

Gelada A-AAAACAACT**G**GGTAGGGTGA

Ancestral sequences 11 A-AATACAACT**G**GGTAGGGTGA

Marmoset A-AATACAGCT**G**GGTAGATTGA

**rs11174811(A/C)**

Human TCATGCTTTT**C**TTGACCAATA

Ancestral sequences 1 TCATGCTTTT**C**TTGACCAATA

Bonobo TCATGCTTTT**C**TTGACCAATA

Ancestral sequences 2 TCATGCTTTT**C**TTGACCAATA

Chimpanzee TCATGCTTTT**C**TTGACCAATA

Ancestral sequences 3 TCATGCTTTT**C**TTGACCAATA

Gorilla TCATGCTTTT**C**TTGACCAATA

Ancestral sequences 4 TCATGCTTTT**C**TTGACCAATA

Orangutan TCATGCTTTT**C**TTGACCAATA

Ancestral sequences 5 TCATGCTTTT**C**TTGACCAATA

Gibbon TCATACTTTT**C**TTGACCAATA

Ancestral sequences 6 TCATGCTTTT**C**TTGACCAATA

Vervet-AGM TCATGCTTTT**C**TTTACCAGTA

Ancestral sequences 7 TCATGCTTTT**C**TTTACCAGTA

Crab-eating macaque TCATGCTTTT**C**TTTACCAGTA

Ancestral sequences 8 TCATGCTTTT**C**TTTACCAGTA

Macaque TCATGCTTTT**C**TTTACCAGTA

Ancestral sequences 9 TCATGCTTTT**C**TTTACCAGTA

Olive baboon TCATGCTTTT**C**TTTACCAGTA

Ancestral sequences 10 TCATGCTTTT**C**TTTACCAGTA

Gelada TCATGCTTTT**C**TTTACCAGTA

Ancestral sequences 11 TCATGCTTTT**C**TTGACCAATA

Marmoset TTGTGCTTTC**C**TTGACCAATA

Ancestral sequences 12 TCATGCTTTT**C**TTGACCAATA

Mouse Lemur TCATGCTTTG**T**GTGGCCAATA

**rs3021529(A/G)**

Human TTCCCACAGC**G**GGGATGGCGG

Ancestral sequences 1 TTCCCACAGC**G**GGGATGGCGG

Bonobo TTCCCACAGC**G**GGGATGGCGG

Ancestral sequences 2 TTCCCACAGC**G**GGGATGGCGG

Chimpanzee TTCCCACAGC**G**GGGATGGCGG

Ancestral sequences 3 TTCCCACAGC**G**GGGATGGCGG

Gorilla TTCCCACAGC**G**GTGATGGCGG

Ancestral sequences 4 TTCCCACAGC**G**GGGATGGCGG

Orangutan TTCCCACAGC**G**GGGATGGC[G](https://useast.ensembl.org/Pongo_abelii/ZMenu/TextSequence?db=core;factorytype=Location;r=12:63151400-63152400;vdb=variation;vf=2280975)G

Ancestral sequences 5 TTCCCACAGC**G**GGGATGGCGG

Gibbon TTCCCACAGC**G**GGGATGGCGG

Ancestral sequences 6 TTCCCACAGC**G**GGGATGGCGG

Vervet-AGM TTCCCACAGC**G**GGGATGGCGG

Ancestral sequences 7 TTCCCACAGC**G**GGGATGGCGG

Crab-eating macaque TTCCCACAGC**G**GGGATGGCGG

Ancestral sequences 8 TTCCCACAGC**G**GGGATGGCGG

Macaque TTCCCACAGC**G**[G](https://useast.ensembl.org/Macaca_mulatta/ZMenu/TextSequence?db=core;factorytype=Location;r=12:63151400-63152400;vdb=variation;vf=6298353)GGATGGCGG

Ancestral sequences 9 TTCCCACAGC**G**GGGATGGCGG

Ancestral sequences 10 TTCCCACAGC**G**GGGATGGCGG

Gelada TTCCCACAGC**G**GGGATGGCGG

Ancestral sequences 11 TTCCCACAGC**G**GGGATGGCGG

Marmoset TTCCCATAGC**G**GGGATAGCGG

Ancestral sequences 12 TTCCCACAGC**G**GGGATGGCGG

Mouse Lemur TTCCCACAGC**G**GAAATGGCGG

**rs10877969(T/C)**

Human CTTT---GTT-TAA**T**CCATATAGTT

Ancestral sequences 1 CTTT---TTT-TAA**C**CCATATAGTT

Bonobo CTTT---TTT-TAA**C**CCATAGAGTT

Ancestral sequences 2 CTTT---TTT-TAA**C**CCATAGAGTT

Chimpanzee CTTT---TTT-TAA**C**CCATAGAGTT

Ancestral sequences 3 CTTT---TTT-TAA**C**CCATATAGTT

Gorilla CTTT---TTT-TAA**C**CCATATAGTT

Ancestral sequences 4 CTTTTTTTTT-TAA**C**CCATATAGTT

Orangutan CTTTTTTTTT-TAA**C**CCATATAGTT

Ancestral sequences 5 CTTTTTTTTT-AAA**C**CCATATAGTT

Gibbon CTTTTTTTTT-AAA**C**CCATA-TGTT

Ancestral sequences 6 CTTT---TTT-AAA**C**CCATATAGTT

Vervet-AGM CTTT---TTTTAAA**G**CCATATAGTC

Ancestral sequences 7 CTTT---TTTAAAA**G**CCATATAGTT

Crab-eating macaque CTTT---TTTAAAA**G**CCATATAGTT

Ancestral sequences 8 CTTT---TTTAAAA**G**CCATATAGTT

Macaque CTTT---TTTAAAA**G**CCATATAGTT

Ancestral sequences 9 CTTT---TTTAAAA**G**CCATATAGTT

Olive baboon CTTT---TTTAAAA**G**CCATATAGTT

Ancestral sequences 10 CTTT---TTTAAAA**G**CCATATAGTT

Gelada CTTT---TTTAAAA**G**CCATGTAGTT

Ancestral sequences 11 CTTT----TT-AAA**C**CCATATAGTT

Marmoset CTTT----TT-AAA**C**CCATATAGTT

**rs3759292(G/A)**

Human ACTATTAC-AT**A**-T[G](https://useast.ensembl.org/Homo_sapiens/ZMenu/TextSequence?db=core;factorytype=Location;r=12:63153033-63154033;vdb=variation;vf=492912712)AGGCA[C](https://useast.ensembl.org/Homo_sapiens/ZMenu/TextSequence?db=core;factorytype=Location;r=12:63153033-63154033;vdb=variation;vf=627217685)TA

Ancestral sequences 1 ACTATTAC-AT**A**-TGAGGCACTA

Bonobo ACTATTAC-AT**A**-TGAGGCACTA

Ancestral sequences 2 ACTATTAC-AT**A**-TGAGGCACTA

Chimpanzee ACTATTAC-AT**A**-TGAGGCACTA

Ancestral sequences 3 ACTATTAC-AT**A**-TGAGGCACTA

Gorilla ACTATTAC-AT**A**-TGAGGCACTA

Ancestral sequences 4 ACTATTACAAT**A**TAGACTCACTA

Orangutan ACTATTACAAT**A**TAGACTCACTA

Ancestral sequences 5 ACTATTACAAT**A**TAGACTCACTA

Gibbon ACCATTACAAT**A**TAGACTCACTA

Ancestral sequences 6 ACTATTACAAT**A**TAGACTCACTA

Vervet-AGM ACTATTACAAT**A**TAGACTCACTA

Ancestral sequences 7 ACTATTACAAT**A**TAGACTCACTA

Crab-eating macaque ACTATTACAAT**A**TAGATTCACTA

Ancestral sequences 8 ACTATTACAAT**A**TAGATTCACTA

Macaque ACTATTACAAT**A**TAGA[T](https://useast.ensembl.org/Macaca_mulatta/ZMenu/TextSequence?db=core;factorytype=Location;r=12:63153033-63154033;vdb=variation;vf=6298394)TCACTA

Ancestral sequences 9 ACTATTACAAT**A**TAGACTCACTA

Olive baboon ACTATTACAAT**A**TAGACTCACTA

Ancestral sequences 10 ACTATTACAAT**A**TAGACTCACTA

Gelada ACTATTACAAT**A**TAGACTCACTA

Ancestral sequences 11 ACTATTACAAT**A**TAGACTCACTA

Marmoset ACCATTACAAT**A**CAGACTTACTA

**VTR1B**

**rs28676508(T/C)**

Human [T](https://useast.ensembl.org/Homo_sapiens/ZMenu/TextSequence?db=core;factorytype=Location;r=1:206109845-206110845;vdb=variation;vf=27045367)[GG](https://useast.ensembl.org/Homo_sapiens/ZMenu/TextSequence?db=core;factorytype=Location;r=1:206109845-206110845;vdb=variation;vf=26529267)[C](https://useast.ensembl.org/Homo_sapiens/ZMenu/TextSequence?db=core;factorytype=Location;r=1:206109845-206110845;vdb=variation;vf=26529267;vf=5107748)[G](https://useast.ensembl.org/Homo_sapiens/ZMenu/TextSequence?db=core;factorytype=Location;r=1:206109845-206110845;vdb=variation;vf=26529267;vf=1372981)[G](https://useast.ensembl.org/Homo_sapiens/ZMenu/TextSequence?db=core;factorytype=Location;r=1:206109845-206110845;vdb=variation;vf=26529267)[C](https://useast.ensembl.org/Homo_sapiens/ZMenu/TextSequence?db=core;factorytype=Location;r=1:206109845-206110845;vdb=variation;vf=26529267;vf=27110211)[T](https://useast.ensembl.org/Homo_sapiens/ZMenu/TextSequence?db=core;factorytype=Location;r=1:206109845-206110845;vdb=variation;vf=26529267)**C**[G](https://useast.ensembl.org/Homo_sapiens/ZMenu/TextSequence?db=core;factorytype=Location;r=1:206109845-206110845;vdb=variation;vf=26529267;vf=26993446)[A](https://useast.ensembl.org/Homo_sapiens/ZMenu/TextSequence?db=core;factorytype=Location;r=1:206109845-206110845;vdb=variation;vf=26529267;vf=26563355)[GA](https://useast.ensembl.org/Homo_sapiens/ZMenu/TextSequence?db=core;factorytype=Location;r=1:206109845-206110845;vdb=variation;vf=26529267)[G](https://useast.ensembl.org/Homo_sapiens/ZMenu/TextSequence?db=core;factorytype=Location;r=1:206109845-206110845;vdb=variation;vf=26529267;vf=27102519)[G](https://useast.ensembl.org/Homo_sapiens/ZMenu/TextSequence?db=core;factorytype=Location;r=1:206109845-206110845;vdb=variation;vf=26529267;vf=27025118)[T](https://useast.ensembl.org/Homo_sapiens/ZMenu/TextSequence?db=core;factorytype=Location;r=1:206109845-206110845;vdb=variation;vf=26588701)G[C](https://useast.ensembl.org/Homo_sapiens/ZMenu/TextSequence?db=core;factorytype=Location;r=1:206109845-206110845;vdb=variation;vf=27221109;vf=27145776)GC

Ancestral sequences 1 TGTGGCGGCT**C**GAGAGGCTGC

Bonobo TGTGGCGGCT**C**GAGAGGCTGC

Ancestral sequences 2 TGTGGCGGCT**C**GAGAGGCTGC

Chimpanzee TGTGGCGGCT**C**GAGAGGCTGC

Ancestral sequences 3 TGTGGCGGCT**C**GAGAGGCTGC

Gorilla TGTGGCGGCT**C**GAGAGGCTGC

Ancestral sequences 4 TGTGGCGGCT**C**GAGAGGCTGC

Orangutan TGTGGCGGCT**C**GAGAGGCTGC

Ancestral sequences 5 TGTGGCGGCT**C**GAGAGGCTGC

Gibbon TGTGGCGGCT**C**GAGAGGCTGC

Ancestral sequences 6 TGTGGCGGCT**C**GAGAGGCTGC

Vervet-AGM TGTGGCGGCT**C**GAAAGGCTGC

Ancestral sequences 7 TGTGGCGGCT**C**GAGAGGCTGC

Crab-eating macaque TGTGGCGGCT**C**GAGAGGCTGC

Ancestral sequences 8 TGTGGCGGCT**C**GAGAGGCTGC

Macaque TGTGGCGGCT**C**GAGAGGCTGC

Ancestral sequences 9 TGTGGCGGCT**C**GAGAGGCTGC

Olive baboon TGTGGCGGCT**C**GAGAGGCTGC

Ancestral sequences 10 TGTGGCGGCT**C**GAGAGGCTGC

Gelada TGTGGCGGCT**C**GAGAGGCTGC

Ancestral sequences 11 TGTGGCGGCT**C**GAGAGGCTGC

Marmoset TGTGGCGGCT**C**GAGAGGCTGC

Ancestral sequences 12 TGTGGCGGCT**C**GAGAGGCTGC

Mouse Lemur TGCGGCGGCT**C**GACAGGCTGC

**rs28632197(T/C)**

Human [G](https://useast.ensembl.org/Homo_sapiens/ZMenu/TextSequence?db=core;factorytype=Location;r=1:206109873-206110873;vdb=variation;vf=26651346)AG[C](https://useast.ensembl.org/Homo_sapiens/ZMenu/TextSequence?db=core;factorytype=Location;r=1:206109873-206110873;vdb=variation;vf=26712707)[C](https://useast.ensembl.org/Homo_sapiens/ZMenu/TextSequence?db=core;factorytype=Location;r=1:206109873-206110873;vdb=variation;vf=26712707;vf=107112316)[G](https://useast.ensembl.org/Homo_sapiens/ZMenu/TextSequence?db=core;factorytype=Location;r=1:206109873-206110873;vdb=variation;vf=26712707;vf=7353507)[C](https://useast.ensembl.org/Homo_sapiens/ZMenu/TextSequence?db=core;factorytype=Location;r=1:206109873-206110873;vdb=variation;vf=26712707)[C](https://useast.ensembl.org/Homo_sapiens/ZMenu/TextSequence?db=core;factorytype=Location;r=1:206109873-206110873;vdb=variation;vf=26712707;vf=5967678)[G](https://useast.ensembl.org/Homo_sapiens/ZMenu/TextSequence?db=core;factorytype=Location;r=1:206109873-206110873;vdb=variation;vf=26712707;vf=26892917)[G](https://useast.ensembl.org/Homo_sapiens/ZMenu/TextSequence?db=core;factorytype=Location;r=1:206109873-206110873;vdb=variation;vf=26712707;vf=135736929)**C**[G](https://useast.ensembl.org/Homo_sapiens/ZMenu/TextSequence?db=core;factorytype=Location;r=1:206109873-206110873;vdb=variation;vf=26712707;vf=26926464)[C](https://useast.ensembl.org/Homo_sapiens/ZMenu/TextSequence?db=core;factorytype=Location;r=1:206109873-206110873;vdb=variation;vf=26712707)[A](https://useast.ensembl.org/Homo_sapiens/ZMenu/TextSequence?db=core;factorytype=Location;r=1:206109873-206110873;vdb=variation;vf=26712707;vf=26844884)[T](https://useast.ensembl.org/Homo_sapiens/ZMenu/TextSequence?db=core;factorytype=Location;r=1:206109873-206110873;vdb=variation;vf=26712707;vf=27122188)[C](https://useast.ensembl.org/Homo_sapiens/ZMenu/TextSequence?db=core;factorytype=Location;r=1:206109873-206110873;vdb=variation;vf=26712707;vf=27045969)[CTGGG](https://useast.ensembl.org/Homo_sapiens/ZMenu/TextSequence?db=core;factorytype=Location;r=1:206109873-206110873;vdb=variation;vf=26712707)

Ancestral sequences 1 GAGCCGCCGG**C**GCATCCTGGG

Bonobo GAGCCGCCGG**C**GCATCCTGGG

Ancestral sequences 2 GAGCCGCCGG**C**GCATCCTGGG

Chimpanzee GAGCCGCCGG**C**GCATCCTGGG

Ancestral sequences 3 GAGCCGCCGG**C**GCATCCTGGG

Gorilla GAGCCGCCGG**C**GCATCCTGGG

Ancestral sequences 4 GAGCCGCCGG**C**GCATCCTGGG

Orangutan GAGCCGCCGG**C**GCATCTTGGG

Ancestral sequences 5 GAGCCGCCGG**C**GCATCCTGGG

Gibbon GAGCCGCCGG**C**GCATCCTGGG

Ancestral sequences 6 GAGCCGCCGG**C**GCATCCTGGG

Vervet-AGM GAGCCGCCGG**C**ACATCCTGGG

Ancestral sequences 7 GAGCCGCCGG**C**ACATCCTGGG

Crab-eating macaque GAGCCGCCGG**C**ACATCCTGGG

Ancestral sequences 8 GAGCCGCCGG**C**ACATCCTGGG

Macaque GAGCCGCCGG**C**ACATCCTGGG

Ancestral sequences 9 GAGCCGCCGG**C**ACATCCTGGG

Olive baboon GAGCCGCCGG**C**ACATCCTGGG

Ancestral sequences 10 GAGCCGCCGG**C**ACATCCTGGG

Gelada GAGCCGCCGG**C**ACATCCTGGG

Ancestral sequences 11 GAGCCGCCGG**C**GCATCCGGGG

Marmoset GAGCCGCCGG**C**GCATCCGGGG

Ancestral sequences 12 GAGCCGCCGG**C**GCATCCGGGG

Mouse Lemur GAGCTGCCGG**C**GCATGCGTGG

**rs33985287(T/C)**

Human [C](https://useast.ensembl.org/Homo_sapiens/ZMenu/TextSequence?db=core;factorytype=Location;r=1:206109572-206110572;vdb=variation;vf=117153085)[G](https://useast.ensembl.org/Homo_sapiens/ZMenu/TextSequence?db=core;factorytype=Location;r=1:206109572-206110572;vdb=variation;vf=28166652)[C](https://useast.ensembl.org/Homo_sapiens/ZMenu/TextSequence?db=core;factorytype=Location;r=1:206109572-206110572;vdb=variation;vf=150385143)TC[C](https://useast.ensembl.org/Homo_sapiens/ZMenu/TextSequence?db=core;factorytype=Location;r=1:206109572-206110572;vdb=variation;vf=27597790)[G](https://useast.ensembl.org/Homo_sapiens/ZMenu/TextSequence?db=core;factorytype=Location;r=1:206109572-206110572;vdb=variation;vf=137259910)CTT**T**--AG[A](https://useast.ensembl.org/Homo_sapiens/ZMenu/TextSequence?db=core;factorytype=Location;r=1:206109572-206110572;vdb=variation;vf=64297089)CAG[G](https://useast.ensembl.org/Homo_sapiens/ZMenu/TextSequence?db=core;factorytype=Location;r=1:206109572-206110572;vdb=variation;vf=114566099)GCT

Ancestral sequences 1 CGCTCCGCTT**C**--AGACAGGGCT

Bonobo CGCTCCGCTT**C**--AGACAGGGCT

Ancestral sequences 2 CGCTCCGCTT**C**--AGACAGGGCT

Chimpanzee CGCTCCGCTT**C**--AGACAGGGCT

Ancestral sequences 3 CACTCCGCTT**C**--AGACAGGGCT

Gorilla CACTCCGCTT**C**--AGACAGGGCT

Ancestral sequences 4 CACTCCGCTT**C**--AGACAGGGCT

Orangutan CACTCCGCTT**C**--AGACAGGGCT

Ancestral sequences 5 CACTCCGCTT**C**--AGACAGGGCT

Gibbon CACTCCGCTT**C**--AGACAGGGCT

Ancestral sequences 6 CACCCCGCTT**C**--AGACAGGGCT

Vervet-AGM CACCCTGCTT**C**--AGACAGGGCT

Ancestral sequences 7 CACCCCGCTT**C**--AGACAGGGCT

Crab-eating macaque CACCCCGCTT**C**--AGACAGGGCT

Ancestral sequences 8 CACCCCGCTT**C**--AGACAGGGCT

Macaque CACCCCGCTT**C**--AGAC[A](https://useast.ensembl.org/Macaca_mulatta/ZMenu/TextSequence?db=core;factorytype=Location;r=1:206109572-206110572;vdb=variation;vf=19094829)GGGCT

Ancestral sequences 9 CACCCCGCTT**C**--AGACAGGGCT

Olive baboon CACCCCGCTT**C**--AGACAGGGCT

Ancestral sequences 10 CACCCCGCTT**C**--AGACAGGGCT

Gelada CACCCCGCTT**C**--AGACAGGGCT

Ancestral sequences 11 CACCCCGCTT**C**--AGACAGGGCT

Marmoset CACCCCGCTT**C**--AGACAGGGCT

Ancestral sequences 12 CACTCCGCTT**C**--AGACAGGGCT

Mouse Lemur CACTCTGCCT**C**CTCGGGTGGGAC

## Supplementary Note 2: Association studies

MH variant sites in *OTR* had the highest association with sociality (**Supplementary Table 3**): 72% of the findings were sociality-related (e.g., ‘empathy’, ‘social temperament’, ‘face recognition’); 14% possibly related to sociality (e.g., ‘obsessive compulsion disorder’, ‘depression’); while the remaining 14% was not related to sociality (e.g., ‘bulimia’, ‘overeating’, ‘diabetes’). These differences were significant (Chi-squared test; P < 0.0001). In contrast, variants in *VTR1A* had a weaker association, with 27% being related to sociality (e.g., ‘moral judgement’, ‘aggression’, ‘autism’), 6% possibly related to sociality (e.g., ‘susceptibility for panic disorder’), and 68% to other phenotypes (e.g., ‘nicotine dependence’, ‘heroin addiction’, ‘hypertension’; **Supplementary Table 4**; Chi-squared test; P = 0.0049). Concerning *VTR1B*, 50% of the findings were related to sociality (e.g., ‘emotional empathy’, ‘aggression’), 32% possibly related to sociality (‘bipolar disorder’, ‘psychotic features’), and 17% were not (e.g. ‘elevated body mass index’; **Supplementary Table 5**). Unlike the other two genes, these values for *VTR1B* were not significantly different between groups (Chi-squared test; P = 0.3417). Lastly, the difference between the proportions of the studies related to sociality were significant for *OTR* versus *VTR1A* (Chi-squared test; P < 0.0001), but not significant for *OTR* versus *VTR1B* (Chi-squared test; P = 0.0771) and *VTR1A* versus *VTR1B* (Chi-squared test; P = 0.1836; **Supplementary Table 6**). We interpret these findings as evidence for a higher functional association to sociality for *OT**R* over *VTR1A* and *VTR1B*, although we are aware it could be a result of publication bias favoring sociality studies with *OTR.*

Regarding the effect of gender in the associations reported, we found 5 studies with gender-specific associations (3 on OTR^3–5^; 1 on VTR1A^6^; and 1 on VTR1B^7^). However, these effects did not affect the functional associations overall, in a way that made them tip towards males or females. For example, there were cases of the same allele (e.g., *OTR*-rs4686302-T and social connectedness^3^) having opposite effects in males and females; or of different alleles (G/T) on the same site (*OTR*-rs6770632) having the same effect (aggressive behavior) in males (G) and females (T)^5^. In other words, the functional association in these sites to gender remains, although the direction of the effect could differ.

We also understand that oftentimes variants identified in one study are not replicated in other samples^8^, but we were not able to find such example in our literature review. This was the case despite studies having different scopes (some focus on a single site, others on a single gene, a single gene pathway, and others run genome-wide analyses), different methods (e.g., different p value cut-offs, different sequencing technologies), different clinical populations, or different populations from different ethnical or national groups.

**Supplementary Tables 7-9**

| RsID | Alleles | Effect | Trial Sample |
| --- | --- | --- | --- |
| rs2228485 | A G | Loneliness^9^ASD^10^Emotion recognition^11^ | 285 (102M, 187F)195 (Chinese: 174 M, 21 F)76 (39 M, 37 F) |
| rs237897 | GA | ASD^12^Altruism^13^Lower self-reported betrayal levels^14^Social connectedness^3^Theory of Mind^15^ | 152 (128 M, 24 F)203 (101 M, 102 F)165 (83 M, 82 F)11.000 (4.146 M, 6.869 F)350 (182 M, 168 F) |
| rs11131149 | GA | Theory of Mind, higher levels of social cognition^15^Depressive mood^16^Lower levels of social cognition^15^ | 350 (182 M, 168 F)493 (Japan.: 307 M, 186 F)350 (182 M, 168 F) |
| rs59190448 | AG | Anxiety, stress and depression risk^17^ | 653 (Caucasian: 347 M, 306 F) |
| rs13316193 | TC | ASD^12^ Depressive mood^16^ Poor empathic communication^18^ Empathy^4^ Poor social skills^19^ Greater cooperation and comforting^20^ ADHD^21^Face emotion recognition (ADHD)^22^ | 152 (128 M, 24 F)493 (Japan.: 307 M, 186 F)120 (60 M, 60 F)101 (Chinese: 46 M, 45 F)112 (104 M, 8 F)422 (Chinese M)276 (145 M, 131 F)151 |
| rs9872310 | AG | Altruism^13^ASD^12^ | 203 (101 M, 102 F)152 (128 M, 24 F) |
| rs4686302 | CT | Better perspective taking skills^4^Face emotion recognition (ADHD)^22^Social connectedness in men, opposite in women^3^ | 101 (Chinese: 46 M, 45 F)15111.000 (4.146 M, 6.869 F) |
| rs237888 | CT | IQ and VABS scores^12^Altruism^13^Greater impairment of ASD^23^Methylation of CpG sites linked to abuse and psychiatric symptoms^24^ | 152 (128 M, 24 F)203 (101 M, 102 F)1002 (841 M, 161 F)393 (African/American: 115 M, 278 F) |
| rs6770632 | AG | Aggression^5^VABS scores^12^ | 236 (162 M, 74 F)152 (128 M, 24 F) |
| rs237885 | GT | Altruism^13^ASD^25^Schizophrenia^26^Callous/unemotional traits^5^Higher risk of aggression^27^ | 203 (101 M, 102 F)282 (Japan.: 240 M, 42 F)145236 (162 M, 74 F)488 (Chinese M) |
| rs1042778 | GT | Lower levels of OT in plasma, diminished parental care^28^Panic and aggressive behaviors^29^Recovery from low maternal emotional warmth^30^ASD^12^Aggression^5^Prosocial fund allocations in the Dictator Game^13^Panic and aggression in ASD^29^Altruism, comforting behavior^4^Positive emotions after training^31^ | 272 (Israeli-Jewish: 121 M, 151 F)2.341 (1.053 M, 1.288 F)152 (128 M, 24 F)236 (162 M, 74 F)203 (101 M, 102 F)209 (170 M, 39 F)101 (Chinese: 46 M, 45 F)122 (33 M, 89 F) |
| rs237911 | AG | ASD^10,25^ | 195 (Chin.: 174 M, 21 F), 282 (Japan.: 240 M, 42 F) |
| rs2254298 | GA | Lower communication^18^Variation in empathy^4^Methylation at cg11589699 (increased depression and anxiety)^24^Less sensitive parenting and lower plasma OXT^28^Higher positive affect^26^Lower scores in depressive temperament^16^Higher levels of Retrospective Self-Report of Inhibition and Adult Separation Anxiety^32^Smaller left amygdala^33^ASD^10,25^Lower levels of emotion recognition and resilience scores^34^Increased amygdala volume^35^Fewer social deficits in ADHD, more social deficits in ASD^36^Lower serum OT in ASD patients^37^Positive parenting behavior, physically controlling behavior^38^Responsive to adversity in Borderline Personality Disorder^39^High levels of physical aggression^40^ | 120 (60 M, 60 F)101 (Chinese: 46 M, 45 F)393 (African/American: 115 M, 278 F)272 (Israeli-Jewish: 121 M, 151 F)145493 (Japan.: 307 M, 186 F)93 (Caucas.: 25 M, 68 F)410 (211 M, 199 F)195 (Chin.: 174 M, 21 F), 282 (Japan.: 240 M, 42 F)264 (Korean: 137 M, 127 F)55 (21 M, 34 F)276 (ADHD: 215 M, 61 F),341 (ASD: 268 M, 73 F)55 (43 M, 12 F)157 (F)148 (F)197 (Chinese: 54 M, 143 F) |
| rs53576 | GA | Bulimia Nerviosa^41^Diminished stress^42^Separation anxiety^43^Weak social cognition in ADHD^19^ASD^10,12^Empathy^44,45^Lower psychological resources^46^Social connectedness (women)^3^ | 159 (Korean, F)194 (M)185 (55 M, 130 F)112 (104 M, 8 F)195 (Chin.:174 M, 21 F), 152 (128 M, 24 F)50 (F), 192 (79 M, 113 F)326 (127 M, 199 F)11.000 (4.146 M, 6.869 F) |
| rs2268490 | CT | Altruism^13^Vocal alterations under stress^47^Stress-related vocal symptoms and higher cortisol levels^47^ | 203 (101 M, 102 F)657 (Finnish: 219 M, 438 F) |
| rs2268493 | T | ASD^48–51^Negative scores in social tasks in schizophrenia^52^ADHD^53^Depressive temperament^16^ | 2.333, 210 (173 M, 37 F), 118 (Caucas.: 74 M, 44 F), 527 (322 M, 205 F)74 (53 M, 21 F)99 (65 M, 34 F)493 (Japan.: 307 M, 186 F) |
| rs237917 | T | Emotion recognition^54^ | 207 (Central European M) |
| rs237889 | CT | Utilitarian answers in dilemmas^55^ASD^12^ | 274 (116 M, 158 F), 322 (139 M, 183 F)152 (128 M, 24 F) |

**Supplementary Table 7:** Literature review results for *OTR* identified Single Nucleotide Polymorphisms (SNPs). For each variant site (Variant/SNP ID), the alleles discussed in each study (‘Alleles’), a short note on the main findings (‘Effect’), and the ‘Trial sample’ used for each study, followed by the number of males (‘M’) and females (‘F’), and nationality/ethnicity details of each sample (when reported), are listed.

| **RsID** | **Alleles** | **Effect** | **Trial Sample** |
| --- | --- | --- | --- |
| rs1042615 | A | ASD^56^ | 224 (Finnish: 175 M, 49 F) |
| rs10784339 | G | Stress reactivity and substance addiction risk^57,58^ | 852 (571 M, 281 F), 398 (359 M, 39 F) |
| rs11174811 | C | Substance addiction risk^57,58^  Higher anxiety levels^59^  Aggression^5^ | 852 (571 M, 281 F), 398 (359 M, 39 F)  1090 (German: 325 M, 725 F) 236 (162 M, 74 F) |
| rs3021529 | G | Addiction^6^ | 1.050 (797 M, 253 F) |
| rs10877969 | A | ASD^60,61^ | 150 (Korean trios), 212 (179 M, 33 F) |

**Supplementary Table 8:** Literature review results for *VTR1A* identified Single Nucleotide Polymorphisms (SNPs). For each variant site (Variant/SNP ID), the allele discussed in each study (‘Alleles’), a short note on the main findings (‘Effect’), and the ‘Trial sample’ used for each study, followed by the number of males (‘M’) and females (‘F’) and nationality/ethnicity details of each sample when reported, are listed.

| **RsID** | **Alleles** | **Effect** | **Trial sample** |
| --- | --- | --- | --- |
| rs28676508 | T | Child onset aggression^62^ | 177 (118 M, 59 F) |
| rs28632197 | T | ASD^49^  Panic disorder^63^ | 210 (173 M, 37 F)  186 (German: 58 M, 128 F) |
| rs33985287 | C | Protects against depressive moods^7^ | 464 (250 M, 214 F) |

**Supplementary Table 9:** Literature review results for *VTR1B* identified Single Nucleotide Polymorphisms (SNPs). For each variant site (Variant/SNP ID), the allele discussed in each study (‘Alleles’), a short note on the main findings (‘Effect’), and the ‘Trial sample’ used for each study, followed by the number of males (‘M’) and females (‘F’) and nationality/ethnicity details of each sample when reported, are listed.

**References**

1. Paten, B. *et al.* Genome-wide nucleotide-level mammalian ancestor reconstruction. *Genome Res.* **18**, 1829–1843 (2008).

2. Zerbino, D. R. *et al.* Ensembl 2018. *Nucleic Acids Res.* **46**, D754–D761 (2018).

3. Chang, S.-C. *et al.* Are genetic variations in OXTR, AVPR1A, and CD38 genes important to social integration? Results from two large US cohorts. *Psychoneuroendocrinology* **39**, 257–268 (2014).

4. Wu, N., Li, Z. & Su, Y. The association between oxytocin receptor gene polymorphism (OXTR) and trait empathy. *J. Affect. Disord.* **138**, 468–472 (2012).

5. Malik, A. I., Zai, C. C., Abu, Z., Nowrouzi, B. & Beitchman, J. H. The role of oxytocin and oxytocin receptor gene variants in childhood-onset aggression. *Genes, Brain Behav.* **11**, 545–551 (2012).

6. Saccone, S. F. & others. Cholinergic nicotinic receptor genes implicated in a nicotine dependence association study targeting 348 candidate genes with 3713 SNPs. *Hum. Mol. Genet.* **16**, 36–49 (2006).

7. Dempster, E. L. Evidence of an Association Between the Vasopressin V1b Receptor Gene (AVPR1B) and Childhood-Onset Mood Disorders. *Arch. Gen. Psychiatry* **64**, 1189 (2007).

8. Border, R. *et al.* No Support for Historical Candidate Gene or Candidate Gene-by-Interaction Hypotheses for Major Depression Across Multiple Large Samples. *Am. J. Psychiatry* **176**, 376–387 (2019).

9. Lucht, M. J. & others. Associations between the oxytocin receptor gene (OXTR) and affect, loneliness and intelligence in normal subjects. *Prog. Neuro-Psychopharmacology Biol. Psychiatry* **33**, 860–866 (2009).

10. Wu, S. & others. Positive association of the oxytocin receptor gene (OXTR) with autism in the Chinese Han population. *Biol. Psychiatry* **58**, 74–77 (2005).

11. Lucht, M. J. & others. Associations between the oxytocin receptor gene (OXTR) and ‘mind-reading’ in humans - An exploratory study. *Nord. J. Psychiatry* **67**, 15–21 (2013).

12. Lerer, E. *et al.* Association between the oxytocin receptor (OXTR) gene and autism: Relationship to Vineland Adaptive Behavior Scales and cognition. *Mol. Psychiatry* **13**, 980–988 (2008).

13. Israel, S. & others. The oxytocin receptor (OXTR) contributes to prosocial fund allocations in the Dictator Game and the social value orientations task. *PLoS One* **4**, (2009).

14. Tabak, B. A., McCullough, M. E., Carver, C. S., Pedersen, E. J. & Cuccaro, M. L. Variation in oxytocin receptor gene (OXTR) polymorphisms is associated with emotional and behavioral reactions to betrayal. *Soc. Cogn. Affect. Neurosci.* **9**, 810–816 (2013).

15. Wade, M., Hoffmann, T. J., Wigg, K. & Jenkins, J. M. Association between the oxytocin receptor ( OXTR ) gene and children ’ s social cognition at 18 months. 603–610 (2014) doi:10.1111/gbb.12148.

16. Kawamura, Y. & others. The association between oxytocin receptor gene (OXTR) polymorphisms and affective temperaments, as measured by TEMPS-A. *J. Affect. Disord.* **127**, 31–37 (2010).

17. Myers, A. J. & others. Variation in the oxytocin receptor gene is associated with increased risk for anxiety, stress and depression in individuals with a history of exposure to early life stress. *J Psychiatr Res* **59**, 93–100 (2014).

18. Schneiderman, I., Kanat-Maymon, Y., Ebstein, R. P. & Feldman, R. Cumulative risk on the oxytocin receptor gene (OXTR) underpins empathic communication difficulties at the first stages of romantic love. *Soc. Cogn. Affect. Neurosci.* **9**, 1524–1529 (2014).

19. Park, J. *et al.* Evidence that genetic variation in the oxytocin receptor (OXTR) gene influences social cognition in ADHD. *Prog. Neuro-Psychopharmacology Biol. Psychiatry* **34**, 697–702 (2010).

20. Wu, N. & Su, Y. Variations in the OXTR Gene and Prosocial Behavior: Moderating Effects of Situational Factors. *Integr. Zool.* (2018).

21. Kang, J. I., Kim, H. W., Kim, C. H., Hwang, E. H. & Kim, S. J. Oxytocin receptor gene polymorphisms exert a modulating effect on the onset age in patients with obsessive-compulsive disorder. *Psychoneuroendocrinology* **86**, 45–52 (2017).

22. Kalyoncu, T., Özbaran, B., Köse, S. & Onay, H. Variation in the oxytocin receptor gene is associated with social cognition and ADHD. *J. Atten. Disord.* 1087054717706757 (2017).

23. Harrison, A. J., Gamsiz, E. D., Berkowitz, I. C., Nagpal, S. & Jerskey, B. A. Genetic variation in the oxytocin receptor gene is associated with a social phenotype in autism spectrum disorders. *Am. J. Med. Genet. Part B Neuropsychiatr. Genet.* **168**, 720–729 (2015).

24. Smearman, E. L. & others. Oxytocin receptor genetic and epigenetic variation: association with child abuse and adult psychiatric symptoms. *Child Dev.* **87**, 122–134 (2016).

25. Liu, X. & others. Association of the oxytocin receptor (OXTR) gene polymorphisms with autism spectrum disorder (ASD) in the Japanese population. *J. Hum. Genet.* **55**, 137–141 (2010).

26. Montag, C. & others. Association between Oxytocin Receptor Gene Polymorphisms and Self-Rated ‘Empathic Concern’ in Schizophrenia. *PLoS One* **7**, (2012).

27. Zhang, Y. *et al.* Genetic variants in oxytocin receptor gene (OXTR) and childhood physical abuse collaborate to modify the risk of aggression in Chinese adolescents. *J. Affect. Disord.* **229**, 105–110 (2018).

28. Feldman, R. & others. Sensitive Parenting Is Associated with Plasma Oxytocin and Polymorphisms in the OXTR and CD38 Genes. *Biol. Psychiatry* **72**, 175–181 (2012).

29. Ribeiro, L. de O. P. *et al.* Evidence for Association Between OXTR Gene and ASD Clinical Phenotypes. *J. Mol. Neurosci.* 1–9 (2018).

30. Dobewall, H. *et al.* Oxytocin receptor gene (OXTR) variant rs1042778 moderates the influence of family environment on changes in perceived social support over time. *J. Affect. Disord.* **235**, 480–488 (2018).

31. Isgett, S. F., Algoe, S. B., Boulton, A. J., Way, B. M. & Fredrickson, B. L. Common variant in OXTR predicts growth in positive emotions from loving-kindness training. *Psychoneuroendocrinology* **73**, 244–251 (2016).

32. Schiele, M. A. *et al.* Oxytocin receptor gene variation, behavioural inhibition, and adult separation anxiety: Role in complicated grief. *World J. Biol. Psychiatry* 1–9 (2018).

33. Uno, Y. *et al.* Inference of the Protokaryotypes of Amniotes and Tetrapods and the Evolutionary Processes of Microchromosomes from Comparative Gene Mapping. *PLoS One* **7**, e53027 (2012).

34. Kim, H. W., Kang, J. I., An, S. K. & Kim, S. J. Oxytocin receptor gene variants are associated with emotion recognition and resilience, but not with false-belief reasoning performance in healthy young Korean volunteers. *CNS Neurosci. Ther.* (2018).

35. Marusak, H. A. *et al.* Amygdala responses to salient social cues vary with oxytocin receptor genotype in youth. *Neuropsychologia* **79**, 1–9 (2015).

36. Baribeau, D. A. *et al.* Oxytocin receptor polymorphisms are differentially associated with social abilities across neurodevelopmental disorders. *Sci. Rep.* **7**, 11618 (2017).

37. Yang, S. *et al.* Serum oxytocin levels and an oxytocin receptor gene polymorphism (rs2254298) indicate social deficits in children and adolescents with autism spectrum disorders. *Front. Neurosci.* **11**, 221 (2017).

38. Tombeau Cost, K. *et al.* Thinking and doing: the effects of dopamine and oxytocin genes and executive function on mothering behaviours. *Genes, Brain Behav.* **16**, 285–295 (2017).

39. Flasbeck, V., Moser, D., Kumsta, R. & Brüne, M. The OXTR single-nucleotide polymorphism rs53576 moderates the impact of childhood maltreatment on empathy for social pain in female participants: evidence for differential susceptibility. *Front. psychiatry* **9**, (2018).

40. Shao, D. *et al.* Effect of the interaction between oxytocin receptor gene polymorphism (rs53576) and stressful life events on aggression in Chinese Han adolescents. *Psychoneuroendocrinology* (2018).

41. Kim, Y.-R., Kim, J.-H., Kim, C.-H., Shin, J. G. & Treasure, J. Association between the Oxytocin Receptor Gene Polymorphism (rs53576) and Bulimia Nervosa. *Eur. Eat. Disord. Rev.* **23**, 171–178 (2015).

42. Chen, F. S. & others. Common oxytocin receptor gene (OXTR) polymorphism and social support interact to reduce stress in humans. *Proc. Natl. Acad. Sci.* **108**, 19937–19942 (2011).

43. Costa, B. & others. Oxytocin receptor polymorphisms and adult attachment style in patients with depression. *Psychoneuroendocrinology* **34**, 1506–1514 (2009).

44. Laursen, H. R. & others. Variation in the oxytocin receptor gene is associated with behavioral and neural correlates of empathic accuracy. *Front. Behav. Neurosci.* **8**, (2014).

45. Rodrigues, S. M., Saslow, L. R., Garcia, N., John, O. P. & Keltner, D. Oxytocin receptor genetic variation relates to empathy and stress reactivity in humans. *Proc. Natl. Acad. Sci.* **106**, 21437–21441 (2009).

46. Saphire-Bernstein, S., Way, B. M., Kim, H. S., Sherman, D. K. & Taylor, S. E. Oxytocin receptor gene (OXTR) is related to psychological resources. *Proc. Natl. Acad. Sci.* **108**, 15118–15122 (2011).

47. Holmqvist Jämsen & others. Associations Between Vocal Symptoms and Genetic Variants in the Oxytocin Receptor and Arginine Vasopressin 1A Receptor Gene. *J. Speech Lang. Hear. Res.* **60**, 1843 (2017).

48. Campbell, D. B. & others. Association of oxytocin receptor (OXTR) gene variants with multiple phenotype domains of autism spectrum disorder. *J. Neurodev. Disord.* **3**, 101–112 (2011).

49. Francis, S. M. *et al.* ASD and genetic associations with receptors for oxytocin and vasopressin—AVPR1A, AVPR1B, and OXTR. *Front. Neurosci.* **10**, 516 (2016).

50. Napoli, A. Di, Warrier, V., Baron-Cohen, S. & Chakrabarti, B. Genetic variation in the oxytocin receptor (OXTR) gene is associated with Asperger Syndrome. *Mol. Autism* **5**, 48 (2014).

51. Yrigollen, C. M. & others. Genes Controlling Affiliative Behavior as Candidate Genes for Autism. *Biol. Psychiatry* **63**, 911–916 (2008).

52. Davis, M. C. & others. Associations between oxytocin receptor genotypes and social cognitive performance in individuals with schizophrenia. *Schizophr. Res.* **159**, 353–357 (2014).

53. Ayaz, A. B. & others. Oxytocin system social function impacts in children with attention-deficit/hyperactivity disorder. *Am. J. Med. Genet. Part B Neuropsychiatr. Genet.* **168**, 609–616 (2015).

54. Chen, F. S. & others. Genetic modulation of oxytocin sensitivity: a pharmacogenetic approach. *Transl. Psychiatry* **5**, e664--e664 (2015).

55. Bernhard, R. M. & others. Variation in the oxytocin receptor gene ( *OXTR*) is associated with differences in moral judgment. *Soc. Cogn. Affect. Neurosci.* nsw103 (2016) doi:10.1093/scan/nsw103.

56. Kantojärvi, K. & others. Association and Promoter Analysis ofAVPR1Ain Finnish Autism Families. *Autism Res.* **8**, 634–639 (2015).

57. Levran, O. & others. Stress-related genes and heroin addiction: A role for a functional FKBP5 haplotype. *Psychoneuroendocrinology* **45**, 67–76 (2014).

58. Maher, B. S. & others. The AVPR1A Gene and Substance Use Disorders: Association, Replication, and Functional Evidence. *Biol. Psychiatry* **70**, 519–527 (2011).

59. Reuter, M., Cooper, A. J., Smillie, L. D., Markett, S. & Montag, C. A new measure for the revised reinforcement sensitivity theory: psychometric criteria and genetic validation. *Front. Syst. Neurosci.* **9**, (2015).

60. Yang, S. Y. & others. Association study between single nucleotide polymorphisms in promoter region of AVPR1A and Korean autism spectrum disorders. *Neurosci. Lett.* **479**, 197–200 (2010).

61. Yang, S. Y. & others. Replicative genetic association study between functional polymorphisms in AVPR1A and social behavior scales of autism spectrum disorder in the Korean population. *Mol. Autism* **8**, (2017).

62. Zai, C. C. *et al.* Possible genetic association between vasopressin receptor 1B and child aggression. *Psychiatry Res.* **200**, 784–788 (2012).

63. Keck, M. E. & others. Combined effects of exonic polymorphisms in CRHR1 and AVPR1B genes in a case/control study for panic disorder. *Am. J. Med. Genet. Part B Neuropsychiatr. Genet.* **147B**, 1196–1204 (2008).
